# Burden and treatment of chronic obstructive pulmonary disease among people using illicit opioids: matched cohort study in England

**DOI:** 10.1136/bmjmed-2022-000215

**Published:** 2022-09-28

**Authors:** Dan Lewer, Sharon Cox, John R Hurst, Prianka Padmanathan, Irene Petersen, Jennifer K Quint

**Affiliations:** 1 Collaborative Centre for Inclusion Health, University College London, London, UK; 2 Institute of Epidemiology and Healthcare, University College London, London, UK; 3 UCL Respiratory, University College London, London, UK; 4 Population Health Sciences, Bristol Medical School, University of Bristol, Bristol, UK; 5 Department of Primary Care and Population Health, University College London, London, UK; 6 National Heart and Lung Institute, Imperial College London, London, UK

**Keywords:** Substance-related disorders, Pulmonary disease, chronic obstructive, Primary health care, Healthcare Disparities, Health services, Epidemiology

## Abstract

**Objective:**

To understand the burden of chronic obstructive pulmonary disease among people who use illicit opioids such as heroin, and evaluate inequalities in treatment.

**Design:**

Cohort study.

**Setting:**

Patients registered at primary care practices in England.

**Participants:**

106 789 patients in the Clinical Practice Research Datalink with illicit opioid use recorded between 2001 and 2018, and a subcohort of 3903 patients with a diagnosis of chronic obstructive pulmonary disease. For both cohorts, the study sampled a comparison group with no history of illicit opioids that was matched by age, sex, and general practice.

**Main outcome measures:**

In the base cohort: diagnosis of chronic obstructive pulmonary disease and death due to the disease. In the subcohort: five treatments (influenza vaccine, pneumococcal vaccine, pulmonary rehabilitation, bronchodilators or corticosteroids, and smoking cessation support) and exacerbations requiring hospital admission.

**Results:**

680 of 106 789 participants died due to chronic obstructive pulmonary disease, representing 5.1% of all cause deaths. Illicit opioid use was associated with 14.59 times (95% confidence interval 12.28 to 17.33) the risk of death related to chronic obstructive pulmonary disease, and 5.89 times (5.62 to 6.18) the risk of a diagnosis of the disease. Among patients with a new diagnosis, comorbid illicit opioid use was associated with current smoking, underweight, worse lung function, and more severe breathlessness. After adjusting for these differences, illicit opioids were associated with 1.96 times (1.82 to 2.12) times the risk of exacerbations requiring hospital admission, but not associated with a substantially different probability of the five treatments.

**Conclusions:**

Death due to chronic obstructive pulmonary disease is about 15 times more common among people who use illicit opioids than the general population. This inequality does not appear to be explained by differences in treatment, but late diagnosis of the disease among people who use illicit opioids might contribute.

**WHAT IS ALREADY KNOWN ABOUT THIS TOPIC**

- Chronic obstructive pulmonary disease is very common among people who use illicit opioids such as heroin
- The high frequency of the disease is likely due to smoking tobacco and other drugs such as heroin, crack cocaine, and methamphetamine
- Treatments can reduce progression of the disease and improve quality of life, but access to these treatments among patients who use illicit opioids has not been investigated

**WHAT THIS STUDY ADDS**

- In a primary care database in England, the rate of death due to chronic obstructive pulmonary disease was about 15 times higher among patients with a known history of using illicit opioids than among patients with no history, supporting existing evidence of the high frequency of the disease in this group
- Among patients with a new diagnosis of the disease, a history of using illicit opioids was associated with more severe symptoms, which might reflect later diagnosis and missed opportunities for treatment
- Patients with chronic obstructive pulmonary disease who had a history of using illicit opioids did not have substantially different access to treatments for the disease, such as immunisation against respiratory infections and drug treatments; however, this group had double the rate of acute exacerbations compared with those without a history of illicit opioid use

**HOW THIS STUDY MIGHT AFFECT RESEARCH, PRACTICE, OR POLICY**

- Smoking cessation should be prioritised by services that support people who use illicit opioids, such as community drug and alcohol services
- Future research could investigate the effectiveness of partnership models in which primary care and specialist respiratory services work with drug and alcohol services to provide respiratory clinics, with the aim of identifying patients with undiagnosed chronic obstructive pulmonary disease

## Introduction

Chronic obstructive pulmonary disease (COPD) is a heterogeneous respiratory condition characterised by airflow obstruction that is not fully reversible. It causes substantial morbidity and mortality in the general population of most countries,^1^ is strongly associated with older age and tobacco smoking,^2^ and is especially prevalent among people who use illicit opioids such as heroin. Cross sectional spirometry studies in community drug and alcohol services have found the following prevalences of COPD (defined as forced exhaled volume in 1 second less than 70% of forced vital capacity): 91/184 (49%; 95% confidence interval 42% to 57%) among people who smoke heroin in Liverpool (UK),^3^ 260/753 (35%; 31% to 38%) in a larger sample of people who smoke heroin in Liverpool,^4^ 36/119 (30%; 23% to 39%) among patients at opioid agonist treatment clinics in Switzerland,^5^ and a pooled value of 18% (95% predictive interval 1% to 90%) from an international systematic review of COPD prevalence among people who smoke opioids.^6^

An important driver of the high burden of COPD in this population is smoking of tobacco and illicit drugs. Tobacco smoking is very common^7 8^ and the duration and intensity of smoking might also be greater than for an average smoker. Smoking crack cocaine and heroin can cause additional damage to lungs through direct thermal injury, irritation of the airways by particles, and opiate stimulated histamine release.^9–11^ Other possible causes of the high frequency of COPD include secondhand smoke, poor living conditions, and poor nutrition.

Although the need for prevention and treatment of COPD in this population is already clear, there is limited understanding of how care can be improved. Spirometry studies^3–5^ show that people who use opioids are willing to be screened and receive a diagnosis, but treatment is mainly in primary care and patients might not always attend appointments. This study aims to compare primary care patients in England with and without a history of illicit opioid use with regards to the incidence of COPD and the rate of death due to COPD; and the treatment and adverse outcomes after a diagnosis of COPD. Our hypothesis was that patients with a history of illicit opioid use would have higher rates of COPD, poorer access to treatment, and poorer outcomes after diagnosis.

## Methods

We studied matched cohorts of people with and without a history of using illicit opioids. The analysis follows a published protocol.^12^

### Data source

We used data from the Clinical Practice Research Datalink (CPRD) Aurum (version April 2020) and Gold (version January 2019).^13 14^ These two databases contain pseudo-anonymised data from general practices in England covering about 13% and 8% of the population, respectively. Participants were limited to those with linkage to the national Hospital Episode Statistics and Office for National Statistics mortality databases.

Participants with a history of illicit opioids were defined as those with clinical observations such as heroin dependence or prescriptions of opioid agonist treatment (methadone or buprenorphine). A full list of codes and validation is published, showing that this sample has similar characteristics to other samples of people who use illicit opioids.^15^ The entry date was the latest of 1 January 2001, 12 months after entry to CPRD, or the first record of illicit opioid use. The 12 month washout period is designed to avoid the unusual time when individuals join primary care research databases, which is often the date of registration at a general practitioner surgery and is associated with poor health and recording of new or pre-existing health conditions. The exit date was the earliest of death and 30 October 2018. A description of rates of all cause mortality and cause specific mortality in this cohort is available elsewhere.^16^

For each participant, we sampled three patients of the same sex and age (±3 years) and from the same general practice, with no previous records of illicit opioid use. The matched participants were assigned the same cohort entry date as the corresponding participant with a history of opioid use. This design is called exposure density sampling.^17^ We sampled a second comparison group for the subcohort of COPD cases. Cases with a history of illicit opioid use were each matched to five cases with no history of illicit opioids, by age at diagnosis (±3 years), sex, and date of diagnosis (±12 months).

### Definition of chronic obstructive pulmonary disease

New diagnoses of COPD in CPRD Gold were based on a validated code list that has an estimated positive predictive value of 87%.^18^ For CPRD Aurum, we created a similar case definition by searching diagnostic terms using the keywords "copd," "chronic obstruct*," "bronchitis," "emphysema," and then screening the results for terms that matched the CPRD Gold case definition.

### Outcomes for participants with chronic obstructive pulmonary disease

Based on the UK National Institute for Health and Care Excellence's guidance NG115 ("chronic obstructive pulmonary disease in over 16s: diagnosis and management"),^19^ we defined five evidence based treatments for COPD: seasonal influenza vaccination, pneumococcal vaccine, referral for pulmonary rehabilitation, inhaled drug treatment specific for COPD, and support with smoking cessation. Except for seasonal influenza vaccine, we classified participants as receiving the intervention in the 12 months after diagnosis or not (ie, binary outcomes). For seasonal influenza vaccine, we considered each influenza season (1 September to 31 March) after COPD diagnosis separately. Participants with a diagnosis of COPD during a influenza season who received a vaccine before diagnosis were considered as being vaccinated. Lists of prescriptions and clinical codes for each outcome and covariate are provided in the study protocol.^12^ We also defined four adverse outcomes after diagnosis of COPD: exacerbations of COPD requiring hospital admission; unplanned hospital admissions with a primary diagnosis of respiratory disease; all cause death; and death with underlying cause of respiratory disease. Definitions and exclusion criteria for each outcome are provided in online supplemental information.

10.1136/bmjmed-2022-000215.supp1 Supplementary data

### Covariates

For incidence of COPD and death due to COPD in the base cohort, analyses were minimally adjusted (ie, adjusted for age and sex), and then additionally adjusted for smoking status in a mediation analysis. For treatment and adverse outcomes after diagnosis in the subcohort, we chose confounders using a causal model (see diagram in online supplemental information). This model considers the effect of a history of using illicit opioids (or membership of this population) on the outcomes, rather than the direct effects of drug use. An alternative study looking at the direct effects of drugs might consider pathways such as interactions between opioids and COPD drug treatments, or the effect of intoxication on appointment attendance. Instead, this study considers the total effect of a history of illicit opioids on COPD treatment and outcomes, including pathways such as patients’ expectations, stigma among staff, and barriers to healthcare such as homelessness. An example of a question that this study aims to answer is: "when a patient receives a COPD diagnosis from a general practitioner, does the fact the patient uses heroin affect their probability of receiving a pneumococcal vaccine?’"

We adjusted analyses for smoking status; body mass index, using the most recent data in the 12 months before cohort entry; COPD GOLD stage,^20^ based on forced expiratory volume in 1 second (FEV1), expressed as % predicted (ie, spirometry); the UK Medical Research Council (MRC) dyspnoea (breathlessness) scale^21^; and comorbidities, defined as the number of unique ICD-10 (international classification of diseases, 10th revision) chapters from diagnoses made during hospital admissions in the three years before COPD diagnosis, limiting to ICD-10 chapters that represent long-term conditions (2-14 and 17). For descriptive purposes, we also extracted information on the index of multiple deprivation^22^ (a composite measure of neighbourhood characteristics such as crime and employment) per participant.

### Statistical analysis

Firstly, we considered death due to COPD using the base cohort. Following the recommended analysis for studies that use exposure density sampling,^17^ we deduplicated individuals (as the comparison group is sampled with replacement), assigned the earliest cohort entry, and expanded data so that participants had a new observation period when they entered a different age band (defined as 18-24, 25-34, 35-44, 45-54, 55-64, and ≥65 years), or if participants in the comparison group have a record of illicit opioid use. Of 320 367 participants in the comparison group, 1310 (0.4%) had a record of illicit opioid use during follow-up. At the first record of illicit opioid use, these participants started a new observation period with a different illicit opioid status (ie, illicit opioid use was time varying). We used left-truncated Cox proportional hazards model with times relative to 1 January 2001. We then repeated this process for analysis of incident COPD diagnosis, excluding participants with a diagnosis of COPD before cohort entry. The models were first adjusted for age and sex only, and then additionally adjusted for tobacco smoking status as a mediation analysis (ie, to what extent does smoking explain opioid related inequalities in COPD?).

Secondly, we focused on the subcohort of participants with a new diagnosis of COPD and considered treatment and adverse outcomes. We used Poisson regression to estimate the relative risk for each treatment. Each model had a binary dependent variable showing whether the treatment was recorded within 365 days of diagnosis, and independent variables of history of illicit opioid use, prespecified confounders, and an offset for the follow-up time. Censoring was the earliest of death, 365 days after diagnosis, or the end of CPRD follow-up (ie, the final date when primary care data was available). For seasonal influenza vaccines, participants were eligible for a vaccine in each influenza season after diagnosis and had a new follow-up period starting on 1 September each year. We included each season as a separate observation period, and used a mixed Poisson model with random intercepts for the individual identifier. We then used Cox proportional hazards models to estimate the association between illicit opioid use and each adverse outcome, with censoring at the earliest of death or 30 October 2018. We did a sensitivity analysis restricted to COPD cases with records of current smoking at diagnosis, because never smokers might have different exposures (eg, might have more genetic risk factors for COPD) and disease phenotypes, and might be more common among the comparison group.

In preliminary descriptions of the dataset^12^ we identified missing data in smoking status, COPD stage, breathlessness symptoms, and body mass index. We used the R package Amelia^23^ to generate 20 complete datasets by multiple imputation for each analysis. Most analyses used Cox proportional hazards regression, and in these imputation models we included the event indicator and the Nelson-Aalen estimator of the cumulative baseline hazard.^24^ We then conducted analysis on each complete dataset and combined the estimates (eg, log hazard ratios) and standard errors using Rubin’s rule.^25^ Analysis was done using R version 3.6.2.

### Patient and public involvement

This study was designed after workshops with people who use illicit opioids in treatment at South London and Maudsley NHS Foundation Trust. The workshops were conducted as part of DL’s doctoral research and focused on access to general health services. Participants highlighted difficulties getting respiratory symptoms assessed and treated in primary care. We plan to communicate these findings to relevant patient groups and stakeholders.

## Results

### Characteristics of participants

The base cohort included 106 789 participants with a history of using illicit opioids and 320 367 matched participants. The median age at study entry was 35.1 years and 73 791 (69.1%) participants were male. Participants with a history of using illicit opioids were more likely to be current smokers (78.2% (83 486/106 789) *v* 33.7% (107 846/320 367)). Characteristics of the base cohort are shown in the online supplemental information.

**Figure 1:**
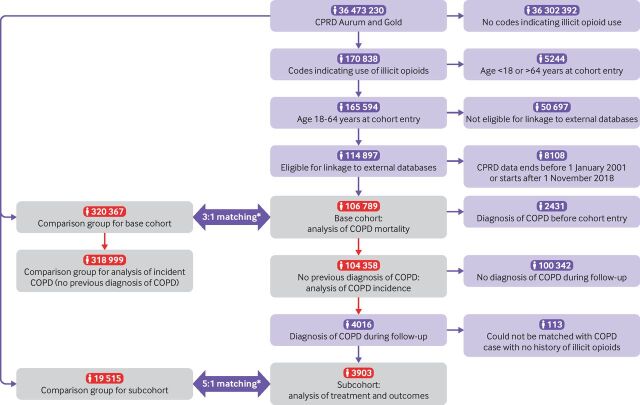
Flowchart showing the derivation of the study sample. Grey shaded boxes with red numbers indicate samples used in analysis. CPRD=Clinical Practice Research Datalink; COPD=chronic obstructive pulmonary disease. *Participants were matched using a process called exposure density sampling, where each participant with a history of using illicit opioids is matched with participants of the same age and sex with no previous record of illicit opioid use, and the two groups are assigned the same cohort entry dates

Figure 1 shows the derivation of the sample, with more detailed diagrams in the online supplemental information. During follow-up, 4016 participants with a history of using illicit opioids received COPD diagnoses; 113 could not be matched with a COPD case with no history of using illicit opioids, and therefore the subcohort included 3903 COPD cases with a history of illicit opioid use and 19 515 matched cases with no history of illicit opioid use.

Table 1 shows characteristics of participants with a new diagnosis of COPD. Median age at diagnosis was 48.8 years in the opioid group and 49.1 years in the comparison group, and 2507 (64.2%) participants were male. Participants with a history of using illicit opioids were more likely to be underweight (11.1% *v* 3.8%), less likely to be overweight or obese (43.9% *v* 60.9%), more likely to be current smokers (86.1% *v* 65.6%), had more comorbidities (median 2 *v* 1), and more severe COPD at diagnosis (63.0% *v* 70.0% median FEV1 as % predicted; and 30.7% *v* 16.8% had MRC breathlessness scores of 2-4).

**Table 1:** Characteristics of participants with a new diagnosis of chronic obstructive pulmonary disease (COPD). Data are number (%) of participants unless stated otherwise

Characteristic	History of using illicit opioids (n=3903)	Comparison group (n=19 515)
Median (IQR) years of observation	3.0 (1.2-5.7)	3.6 (1.7-6.6)
Median (IQR) age at diagnosis (years)	48.8 (43.4-54.4)	49.1 (43.7-54.9)
Sex		
Male	2507 (64.2)	12 535 (64.2)
Female	1396 (35.8)	6980 (35.8)
Body mass index		
Underweight (<18.5)	434 (11.1)	739 (3.8)
Healthy (18.5-25)	1623 (41.6)	6473 (33.2)
Overweight (25-30)	880 (22.5)	5874 (30.1)
Obese (30-40)	689 (17.7)	4982 (25.5)
Severely obese (≥40)	146 (3.7)	1025 (5.3)
Missing	131 (3.4)	422 (2.2)
Median (IQR)	24.2 (20.5-29.1)	26.8 (23.0-31.3)
Tobacco smoking at diagnosis		
Never	61 (1.6)	2276 (11.7)
Ex-smoker	433 (11.1)	4106 (21.0)
Current	3359 (86.1)	12 803 (65.6)
Missing	50 (1.3)	330 (1.7)
No of comorbidities		
0	1258 (32.2)	8522 (43.7)
1-2	1019 (26.1)	5465 (28.0)
3-4	893 (22.9)	3371 (17.3)
5-7	580 (14.9)	1820 (9.3)
≥8	153 (3.9)	337 (1.7)
Median (IQR)	2 (0-4)	1 (0-3)
COPD GOLD spirometry stage: forced exhaled volume in 1 second (FEV1) (% predicted)		
Mild (≥80%)	611 (15.7)	4476 (22.9)
Moderate (50-80%)	1298 (33.3)	7654 (39.2)
Severe (30-50%)	605 (15.5)	1966 (10.1)
Very severe (<30%)	191 (4.9)	340 (1.7)
Missing	1198 (30.7)	5079 (26.0)
Median (IQR)	63.0 (46.0-78.0)	70.0 (57.0-83.0)
MRC dyspnoea scale		
Grade 0 (least severe)	331 (8.5)	4199 (21.5)
Grade 1	957 (24.5)	5278 (27.0)
Grade 2	747 (19.1)	2384 (12.2)
Grade 3	377 (9.7)	783 (4.0)
Grade 4 (most severe)	73 (1.9)	114 (0.6)
Missing	1418 (36.3)	6757 (34.6)
Index of multiple deprivation*		
1 (least deprived)	187 (4.8)	2109 (10.8)
2	304 (7.8)	2866 (14.7)
3	520 (13.3)	3511 (18.0)
4	937 (24.0)	4687 (24.0)
5 (most deprived)	1952 (50.0)	6325 (32.4)
Missing	3 (0.1)	17 (0.1)

IQR=interquartile range; MRC=UK Medical Research Council.

*Participants are matched by general practitioner surgery. Different deprivation scores indicate that participants in the opioid group disproportionately live in poor neighbourhoods around the same surgeries.

### Death and diagnosis

Among 106 789 participants with a history of using illicit opioids, 680 (0.6%) died with an underlying cause of COPD over a median 8.7 years of follow-up (table 2). COPD was the underlying cause in 680 (5.1%) of 13 213 all cause deaths in the cohort. In the comparison group (n=320 367), 160 (0.05%) died with an underlying cause of COPD over a median 9.5 years of follow-up. The Cox proportional hazards model showed that participants with a history of illicit opioids had 14.59 times (95% confidence interval 12.28 to 17.33) the hazard of death with an underlying cause of COPD. After adjustment for smoking status at cohort entry, the hazard ratio was 8.81 (7.36 to 10.54).

**Table 2:** Rates of death and diagnosis of chronic obstructive pulmonary disease (COPD) in base cohort

	Death with underlying cause of COPD	New diagnosis of COPD
**Group with history of illicit opioid use**		
No of participants at baseline	106 789	104 358*
Median (IQR) follow-up per participant (years)	8.7 (4.3-13.5)	3.3 (1.3-7.2)
No of events (ie, death or COPD diagnosis)	680	4016
Rate (95% CI) of events per 100 000 person years	71 (66 to 76)	799 (774 to 824)
**Comparison group (matched by age, sex, and general practice)**		
No of participants at baseline	320 367	318,999*
Median (IQR) years of follow-up per participant (years)	9.5 (5.0-14.4)	5.6 (2.5-10.4)
No of events	160	3051
Rate (95% CI) of events per 100 000 person years	5.6 (4.8 to 6.5)	153 (147 to 158)
**Hazard ratio (95% CI**)		
Adjusted for age and sex only	14.59 (12.28 to 17.33)	5.89 (5.62 to 6.18)
Additionally adjusted for smoking status at cohort entry	8.81 (7.36 to 10.54)	3.29 (3.13 to 3.46)

IQR=interquartile range; CI=confidence interval.

*Excluding participants with a diagnosis of COPD before cohort entry (ie, excluding participants with prevalent COPD).

Among 106 789 patients with a history of using illicit opioids, 2431 (2.3%) had a diagnosis of COPD before joining the cohort, compared with 1368 (0.4%) of 320 367 in the comparison group. During a median 3.3 years of follow-up, 4016 participants with a history of using illicit opioids received a COPD diagnosis, versus 3051 participants in the comparison group over a median of 5.6 years of follow-up. The Cox proportional hazards model showed that participants with a history of illicit opioid use had 5.89 times (95% confidence interval 5.62 to 6.18) the hazard of a new COPD diagnosis. After adjustment for smoking, the hazard was 3.29 (3.13 to 3.46). Kaplan-Meier curves and detailed results from the Cox proportional hazards models are shown in online supplemental information).

### Outcomes after a new diagnosis of chronic obstructive pulmonary disease

The proportions of participants with COPD receiving treatment were similar for those using illicit opioids and for those in the comparison group (table 3). The absolute proportion receiving each treatment varied, with higher proportions for drug treatments (bronchodilators and corticosteroids) and lower proportions for pulmonary rehabilitation referral, pneumococcal vaccine, and smoking cessation support.

**Figure 2:**
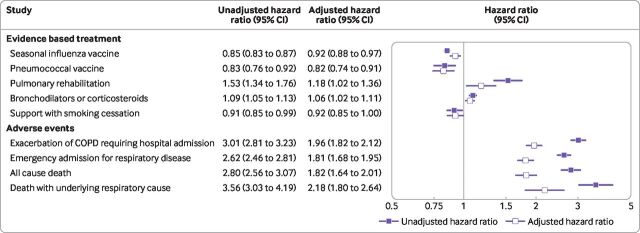
Hazard ratios of evidence based treatment and adverse events after diagnosis of chronic obstructive pulmonary disease (COPD), comparing participants with a history of illicit opioid use to those without. Unadjusted hazard ratios are adjusted for age and sex only. Adjusted hazard ratios are additionally adjusted for smoking status, COPD GOLD group (FEV1/predicted), UK Medical Research Council's dyspnoea/breathlessness scale, and body mass index

Poisson regression showed that illicit opioid use was associated with lower probability of seasonal influenza vaccine and pneumococcal vaccine, and higher probability of pulmonary rehabilitation referral and bronchodilators or corticosteroids. However, the associations were small and confidence intervals were close to a ratio of one (ie, no association). Adjusting for smoking status and disease severity in Poisson regression did not substantially change these associations (figure 2).

**Table 3:** Proportions of participants with a new diagnosis of chronic obstructive pulmonary disease (COPD) receiving treatments, by study group. Data show number of participants eligible for each intervention and the number (%) receiving each intervention within 12 months of diagnosis

COPD treatment	Group with history of illicit opioid use	Comparison group
Seasonal influenza vaccine (years after COPD diagnosis)		
1	1798/3833 (46.9)	8258/16 476 (50.1)
2	1629/3167 (51.4)	8056/14 473 (55.7)
3	1302/2547 (51.1)	6812/12 055 (56.5)
4	1039/2007 (51.8)	5792/10 021 (57.8)
≥5	3447/6332 (54.4)	21 942/35 861 (61.2)
Pneumococcal vaccine	467/3203 (14.6)	2732/15 700 (17.4)
Pulmonary rehabilitation referral	270/3903 (6.9)	880/19 515 (4.5)
Bronchodilators or corticosteroids	3164/3318 (81.1)	14 559/16 590 (74.6)
Smoking cessation support	672/2855 (23.5)	2815/11 027 (25.5)

Participants with a history of illicit opioids before COPD diagnosis had about three times the risk of each adverse outcome (exacerbations requiring hospital admission and death). In Cox proportional hazards models adjusting for smoking status and disease severity, these associations were partially reduced, and participants with a history of illicit opioids had about double the adjusted hazard of each outcome. In sensitivity analysis restricted to participants with records of current smoking at diagnosis, associations between illicit opioid use and outcomes after diagnosis were very similar to the main results (online supplemental information).

## Discussion

### Principal findings

In this sample of primary care patients in England, individuals with a history of illicit opioid use had about six times the risk of new COPD diagnosis and about 15 times the risk of death with an underlying cause of COPD. Among patients diagnosed with COPD, those with a history of using illicit opioids had more severe disease, potentially reflecting later diagnosis. We found no large inequalities in treatment after diagnosis, although illicit opioids were associated with substantially higher frequency of exacerbations requiring hospital admission and death, even after adjusting for disease severity at diagnosis.

### Interpretation of results

Qualitative research has found that people who use illicit opioids have barriers to health services. These include: stigmatising attitudes among healthcare staff, including perceptions that patients who use illicit drugs have poor motivation or are to blame for their health problems^26 27^; barriers to attending appointments, such as transport costs and competing priorities such as finding enough food or money for the day^26 27^; clinicians attributing coughing and other symptoms to drug use, sometimes known as diagnostic overshadowing^28^; and delaying help seeking owing to normalisation of pain and fear of stigma.^29^ These barriers could cause later diagnosis of COPD among people who use illicit opioids. In the present study, patients with a history of illicit opioid use had considerably worse symptoms at diagnosis than those with no history.

People who use illicit opioids in England are ageing,^30^ and the burden of COPD is likely to increase. Given the large inequality and specific health needs in this population, prevention of COPD will require a dedicated strategy. We discuss three approaches to reducing this inequality.

#### Improving diagnosis

The results suggest substantial undiagnosed COPD among people who use or have used illicit opioids. Comparing prevalence from studies that use different diagnostic methods is difficult,^2 31^ but cross sectional spirometry studies have found that 30-40% of people who use illicit opioids have COPD,^4 5^ compared with 2% of participants in this study having a diagnosis of COPD at baseline. One approach is providing spirometry in accessible locations such as those already providing harm reduction interventions (eg, needle and syringe programmes and opioid agonist treatment); an approach that has been piloted in Sheffield, Liverpool, and London^3 4 32 33^ and appears acceptable to participants. However, this approach is screening and should meet certain criteria before implementation,^34 35^ including an accessible treatment pathway and robust evaluation. Another approach is to provide education and training to primary care staff, aiming to reduce stigma and diagnostic overshadowing.^28^

#### Improving access to treatment after diagnosis

Contrary to our hypothesis, we did not find evidence of large inequalities in treatment, which suggests that people with a history of using illicit opioids have similar care as other patients once they receive a COPD diagnosis, or reflects selection bias in which people with COPD who use opioids and have this recorded by their general practitioner are a motivated subgroup. However, the results also show low absolute levels of some interventions, both in the opioid and comparison group. A small proportion of patients (about one in 20 in this study) were referred for pulmonary rehabilitation, which has been previously observed.^36 37^ A review of international evidence found that the most common barrier to referral to pulmonary rehabilitation was that clinicians do not understand its benefits, and that training could help.^38^ Patient level reasons for non-attendance include transport, lack of perceived benefit, continued smoking after diagnosis, and depression.^39^ About one in seven patients received a pneumococcal vaccine. Access to the influenza vaccine was better, at about one in two patients. This difference in access might suggest that low access to pneumococcal vaccine is because there is no annual campaign, as there is for influenza, and awareness of eligibility criteria and guidance might be low among general practitioners. Access to influenza vaccines could be improved by ensuring that open access vaccination is available in settings such as pharmacies that provide injecting equipment and opioid agonist treatment. Although the results show that some treatments are rarely provided, they also suggest that the large inequalities in both the frequency and outcomes of COPD are unlikely to be explained by healthcare access after diagnosis.

#### Primary prevention of COPD through smoking cessation

Most instances of COPD in high income countries are caused by tobacco smoking.^1 40^ Smoking among people who use illicit opioids^7^ is likely an important contributor to the high burden of COPD in this population. Among people without COPD, smoking cessation is associated with lower incidence of COPD,^41^ and among people with COPD of any severity it is associated with slower decline in lung function and reduced mortality.^42^ The Global Initiative for Chronic Obstructive Lung Disease advises that stopping smoking is the most effective therapeutic approach for preventing or reducing the progression of COPD.^20^ Historically, smoking cessation has been considered difficult or unrealistic among people who use illicit drugs, and few attempts have been made to reduce the high prevalence of smoking. In 2019-20, only 2.4% of people starting opioid agonist treatment in England who said they smoke tobacco were provided some kind of smoking cessation intervention.^43^ However, randomised controlled trials of traditional smoking cessation aides (such as nicotine replacement therapy, motivational interviewing, and varenicline or bupropion) among people in treatment for substance use have found sustained reductions in smoking.^44^ Qualitative data suggests that e-cigarettes might be more appealing in this population than traditional treatments.^45^ The provision of e-cigarettes in a parallel population of people using homeless day centres is currently being evaluated in a cluster randomised controlled trial^46^ and this study could inform approaches to smoking cessation for people who use heroin and crack cocaine.

### Strengths and limitations of the study

Other studies have estimated COPD prevalence,^3–5^ incidence,^47^ and mortality^48 49^ among people who use illicit opioids, also finding a high health burden related to COPD. To our knowledge, this is the first study investigating treatment in this population. We used a large, representative database that is likely to reflect clinical reality. We used two well known measures of disease severity (COPD GOLD stage^20^ and the MRC dyspnoea scale^21^), which were well recorded with about two thirds of participants having each measurement at the time of diagnosis. The study uses five cost effective and evidence based interventions recommended in UK guidelines (seasonal influenza vaccine, pneumococcal vaccine, pulmonary rehabilitation, inhaled drug treatment, and support with smoking cessation), providing evidence across these interventions that illicit opioid use was not strongly associated with healthcare access after diagnosis.

The study had four key limitations. Firstly, the study could have had selection biases, owing to missing data on COPD cases or opioid exposures. For missing COPD cases, data from Wales suggest that about a third of patients on general practitioners' COPD registers do not have recorded spirometry values^50^ (consistent with missing data in table 1), and that some patients also might not have the diagnostic codes used in our case definition. Missing COPD diagnoses will mean that absolute incidence of diagnosed COPD is underestimated, although we could not identify a reason why this potential limitation would seriously bias our estimate of the association between illicit opioids and COPD incidence. Missing opioid exposures could have biased our estimates of the association between opioid use and treatment. Disclosure of illicit drug use to a general practitioner might be a marker for good engagement with healthcare, and therefore people with COPD who use illicit opioids but who were not identified in our study might have lower rates of treatment.

Secondly, the measurement of smoking is limited to never smoking, ex-smoking, and current tobacco smoking status at cohort entry. Smokers who use illicit opioids are likely to have longer and heavier smoking histories. We found that the association between opioid use and COPD diagnosis or death was partially explained by tobacco smoking status. However, the limited measurement of smoking meant that we were unable to assess the contribution of smoking to inequality in COPD, and the importance of other exposures. In addition, smoking after diagnosis might vary between the opioid and comparison group, which could contribute to the higher rates of acute exacerbations and death after diagnosis. We also did not have data on the route of opioid administration, and particularly whether participants smoke heroin. Therefore, we were unable to investigate the impact of heroin smoking on COPD risk, or make direct comparisons with studies of COPD among heroin smokers.

Thirdly, there could have been unmeasured differences in disease at diagnosis. The higher rate of acute exacerbations in the opioid group suggests residual differences in severity or exposures during follow-up such as ongoing smoking. We measured severity using the COPD GOLD stage and the MRC breathlessness scale. Although these scales are associated with survival probability,^1 51^ their prognostic value could differ between populations. Differences might also relate to different types of disease. For example, a study of COPD among people who smoke heroin showed that emphysema is the dominant phenotype,^11^ so it is possible that patients with COPD and a history of illicit opioid use are more likely to have emphysema than other patients with COPD and no history. The vast majority of patients had generic COPD diagnoses rather than records of a specific phenotype, and we were not able to subclassify COPD phenotypes.

Finally, eligibility and compliance with COPD treatments was difficult to measure. Eligibility was easily determined for support with smoking cessation (current smokers), pneumococcal vaccine (participants without a previous vaccine), and seasonal influenza vaccine (everyone). For COPD drug treatments and pulmonary rehabilitation, the clinical decision to provide treatment is based on additional patient characteristics^19^ and preferences that were not captured in this study—that is, our eligibility definitions might not capture clinical reality. The proportions of patients actually receiving treatments might also vary between the opioid and comparison groups. Our measures of treatment typically capture the first step taken by a clinician, such as a prescription or referral. Vaccinations are usually given immediately and the rate of prescriptions might closely match the rate of administered vaccines, while patients need to pick up inhalers or attend rehabilitation appointments with some potential dropout (which we could not measure).

### Conclusion

Death due to COPD is 15 times more common among people who use or have used illicit opioids than the general population. This inequality does not appear to be explained by differences in treatment after diagnosis, but later diagnosis might contribute. A strategy to prevent and treat COPD among people who use illicit opioids should include more accessible assessment of respiratory problems and prioritisation of smoking cessation by services that support this population.

## Data Availability

Data may be obtained from a third party and are not publicly available. Codelists for the HUPIO cohort on which this analysis is based are publicly available at https://wellcomeopenresearch.org/articles/5-282/v2. We used these codes in primary care data from CPRD. Researchers can use CPRD by applying to the CPRD Research Data Governance Process. Approval is required if access to anonymised patient level data is being requested for research purposes. Details of the application process and conditions of access are provided by CPRD at https://cprd.com/research-applications.
