# Supplementary material for: Burden and treatment of chronic obstructive pulmonary disease among people using illicit opioids: matched cohort study in England
Source: BMJ Med. 2022 Sep 28;1(1):e000215. doi: 10.1136/bmjmed-2022-000215 (PMC9770021; doi:10.1136/bmjmed-2022-000215)
Supplement: Supplementary data [file bmjmed-2022-000215supp001.pdf]

Supplementary information

1. Changes from analysis protocol 2
2. Definition of comparison groups 3
3. SNOMED concepts used to identify COPD cases in CPRD Aurum 5
4. Causal model for selecting confounding variables in analysis of treatment and outcomes after diagnosis of COPD 8
5. Characteristics of participants in the base cohort 9
6. Definitions and exclusions for outcomes after diagnosis of COPD 10
7. Sensitivity analysis of outcomes after COPD diagnosis, restricted to current smokers 11
8. Detailed results of survival analysis 12
9. References for supplementary information 15

Lewer D, *et al. BMJMED* 2022; 1:e000215. doi: 10.1136/bmjmed-2022-000215

1. Changes from analysis protocol

This study followed a published protocol. We made the following changes to the protocol:

a) **Approach to analysis of missing data**. In our protocol,[1] we planned to use a 'missing indicator' method in which missing categorical observations are included in analysis with a separate category of 'missing', and missing numerical observations are included as zero with a second missing indicator category. We chose to use multiple imputation instead (as described in the methods section) because simulation studies have shown that the 'missing indicator' method is likely to be biased.[2,3]

b) **Sensitivity analysis excluding COPD cases who did not have records of current smoking at diagnosis.** For analyses of outcomes after COPD diagnosis, we did an unplanned sensitivity analysis excluding participants who did not have records of current smoking (i.e. we classified as never-smokers, ex-smokers, or had no records of smoking status). We did this analysis because never-smokers with COPD may have unusual exposures and disease types, and were also more common among cases without a history of using illicit opioids.

c) **A matching ratio of 1:3 rather than 1:5 in the 'base cohort'**. In our protocol, we planned to match each participant with a history of using illicit opioids with five patients without a history of using illicit opioids. We reduced this ratio to 1:3 to meet data sharing requirements of the Clinical Practice Research Datalink. As the base cohort was large (106,789 participants with a history of using illicit opioids), this did not affect the power of our analyses. We retained 1:5 matching for the smaller sub-cohort of patients with a COPD diagnosis.

2. Definition of comparison groups

This study used two comparison groups: (1) CPRD patients with no records of illicit opioid use; and (2) COPD cases with no records of illicit opioid use at diagnosis. Both comparison groups are drawn from the entire CRPD database (i.e. the second comparison group is not a subset of the first). This is shown in Figure 1 of the main article.

Comparison groups were sampled from CPRD patients who were unexposed (i.e. had no prior records of illicit opioid use) at the date of cohort entry of the corresponding exposed participant. This process is called ‘exposure density sampling’,[4] and is designed to minimise biases related to the definition of cohort entry. The exposure density sampling procedures are shown in Figure 1 and Figure 2.

Figure 1: Exposure density sampling to create a comparison group of patients without a history of illicit opioid use. Blue crosses represent potential matches from which the unexposed group is sampled

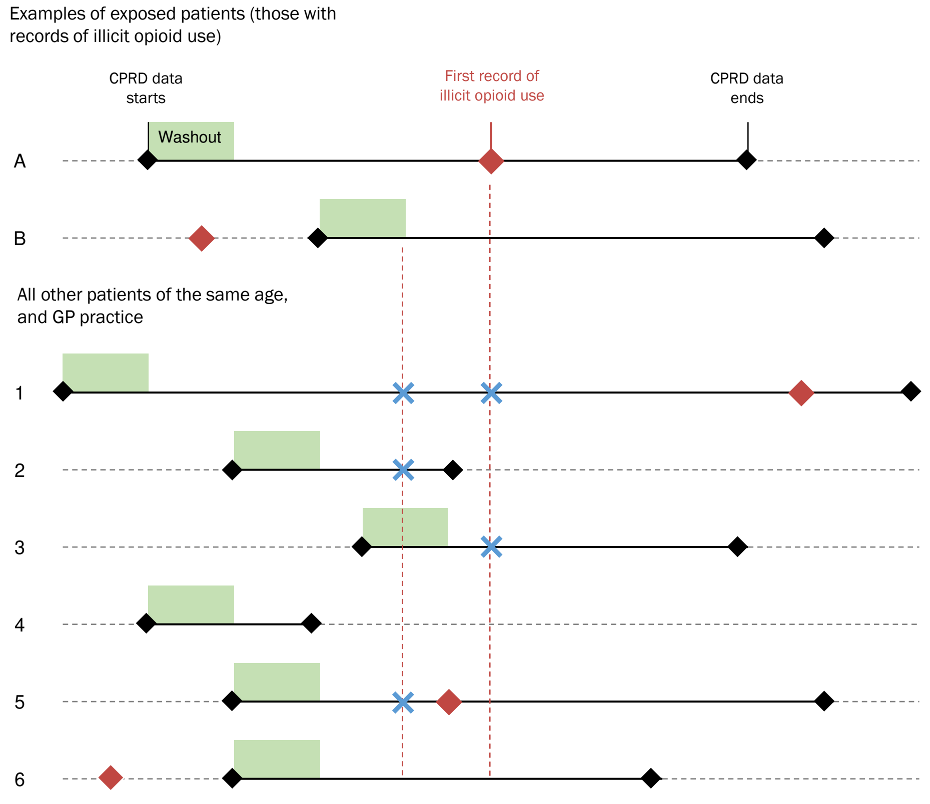

In this figure, participant A joins the cohort when they first use illicit opioids. Participant B has a record of illicit opioid use prior to joining CPRD. The cohort is designed to capture people with a history of illicit opioid use (rather than new opioid use) and therefore participant B is included. They enter the cohort after the washout period, and are matched with patients of the same age and sex who are unexposed on that day. Participant 1 may be matched to both participants A and B, and may therefore be duplicated in the comparison group. Participant 5 may be matched to participant B because they are unexposed at the time when participant B joins the cohort, but will be censored or change exposure status at their first record of illicit opioid use and therefore is not available to be matched with participant A.

Figure 2: Exposure density sampling to create a comparison group of people with a COPD diagnosis but no history of illicit opioid use

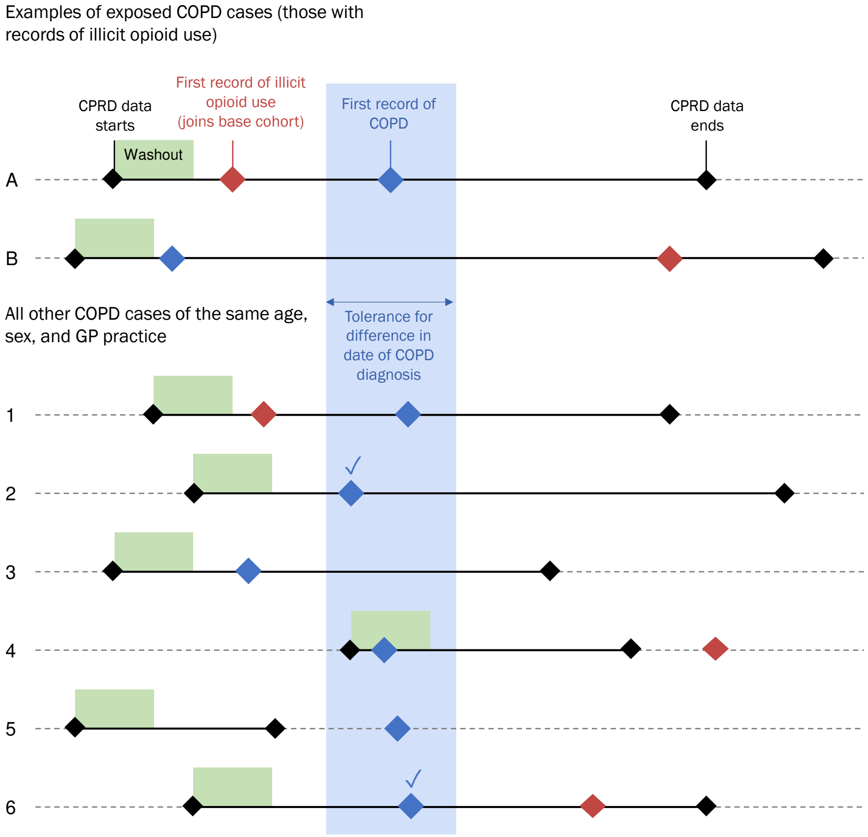

In this example, patient A has a new diagnosis after cohort entry, while patient B has prevalent COPD at cohort entry and is excluded. Ticks represent potential matches from which the unexposed group for patient A is sampled.

Lewer D, *et al. BMJMED* 2022; 1:e000215. doi: 10.1136/bmjmed-2022-000215

3. SNOMED concepts used to identify COPD cases in CPRD Aurum

For patients in CPRD Gold, we used a validated definition of COPD.[5] We used this codelist to create a similar codelist for CPRD Aurum, as described in the methods. The SNOMED codes are shown in Table 1 and are also available at: https://github.com/danlewer/hupio/blob/main/codelists/aurum_copd.csv/. Note that numeric codes are prefixed with an 'x' to prevent errors due to class conversion. Once the codes have been read as strings, this 'x' should be removed.

Table 1: SNOMED codes for COPD diagnosis

MedCodeId	Term
x27096010	Giant bullous emphysema
x105519017	Chronic bronchitis
x123588010	Mucopurulent chronic bronchitis
x216596014	End stage chronic obstructive airways disease
x301456017	Mixed simple and mucopurulent chronic bronchitis
x301460019	Chronic bullous emphysema
x301463017	Segmental bullous emphysema
x301470017	Acute vesicular emphysema
x457168017	Mild chronic obstructive pulmonary disease
x457169013	Moderate chronic obstructive pulmonary disease
x475431013	Chronic obstructive pulmonary disease
x506053014	Purulent chronic bronchitis
x508561017	Simple chronic bronchitis
x1484924013	Chronic obstructive pulmonary disease monitoring
x299001000000116	Chronic obstructive pulmonary disease disturbs sleep
x885281000006118	Mucopurulent chr. bronchitis
x909721000006115	[RFC] Emphysema
x1771201000006116	Chronic obstructive pulmonary disease multidisciplinary review
x1856491000006119	Chronic obstructive pulmonary disease monitoring in primary care
x1856501000006110	Chronic obstructive pulmonary disease monitoring secondary care
x1856571000006116	Chronic obstructive pulmonary disease severity
x1856591000006115	Chronic obstructive pulmonary disease follow-up assessment
x1882421000006113	1 COPD exacerbation in past year
x1882431000006111	3+ COPD exacerbations in past year
x1882441000006118	2 COPD exacerbations in past year
x2219861000000114	Chronic obstructive pulmonary disease rescue pack declined
x2423731000000115	Telehealth chronic obstructive pulmonary disease monitoring
x2010061000006113	Acute non-infective exacerbation of chronic obstructive pulmonary disease
x19421011	Interstitial emphysema
x139979010	Fetid chronic bronchitis
x301444018	Simple chronic bronchitis NOS
x301455018	Obstructive chronic bronchitis NOS
x301458016	Other chronic bronchitis NOS
x301468014	Chronic bullous emphysema NOS
x301477019	Emphysema NOS
x301545019	Chronic obstructive airways disease NOS
x1222334016	Other specified chronic obstructive pulmonary disease
x1222335015	Chronic obstructive pulmonary disease NOS
x553211000006119	Chron obstruct pulmonary dis wth acute exacerbation, unspec
x555461000006119	Chronic obstruct pulmonary dis with acute lower resp infectn
x977891000006112	COPD accident and emergency attendance since last visit
x998281000006115	Multiple COPD emergency hospital admissions
x1704531000006115	COPD patient unsuitable for pulmonary rehab - enh serv admin
x1704541000006113	COPD patient unsuitable for pulmonary rehabilitation
x1783891000006115	Chronic obstructiv pulmonary disease medication optimisation
x1784071000006113	Preferred place of care for next exacerbation of COPD
x1811661000006110	COPD self-management plan agreed
x1885981000006112	Chronic obstructive pulmon dis wr self managem plan declined
x301469018	Other emphysema
x11932351000006110	COPD GOLD group C
x11932361000006112	COPD GOLD group D
x2160051010	Admit COPD emergency
x8312921000006111	COPD (chronic obstructive pulmonary disease) management plan declined
x4733031000006114	Chronic obstructive bronchitis
x2716351000006113	COLD - Chronic obstructive lung disease
x7484341000006112	Optimization of medication for chronic obstructive lung disease
x4781461000006117	Emphysematous bulla
x4733011000006115	COB - Chronic obstructive bronchitis

MedCodeId	Term
x4781421000006111	Acute exacerbation of COPD
x8260631000006112	COPD (chronic obstructive pulmonary disease) self-management plan review
x8294671000006116	COPD (chronic obstructive pulmonary disease) written self management plan declined
x9317331000006113	Asthma-COPD overlap syndrome (ACOS)
x4733021000006111	Obstructive chronic bronchitis
x4732991000006119	Chronic bronchitis with emphysema
x4781431000006114	Acute exacerbation of chronic obstructive pulmonary disease
x3764021000006117	Interstitial emphysema of lung
x8316481000006116	Shared care COPD (chronic obstructive pulmonary disease) monitoring
x8125721000006119	At risk of COPD (chronic obstructive pulmonary disease) exacerbation
x12489801000006116	Admit COPD emergency
x2716321000006116	COPD - Chronic obstructive pulmonary disease
x6763231000006119	Emergency hospital admission for chronic obstructive pulmonary disease
x3921361000006112	Emphysema of lung
x12704691000006117	Interstitial emphysema
x12759361000006112	Chronic obstructive pulmonary disease NOS
x285104011	Obstructive chronic bronchitis
x301539010	Other specified chronic obstructive airways disease
x1880061000006110	COPD management plan declined
x1765681000000110	History of chronic obstructive pulmonary disease
x1573111000000115	Issue of chronic obstructive pulmonary disease rescue pack
x516801000000112	Very severe chronic obstructive pulmonary disease
x1813871000006117	On chronic obstructive pulmonary disease supprtv cre pathway
x11932341000006113	COPD GOLD group B
x977911000006114	Number of COPD exacerbations in past year
x7484351000006114	Optimisation of medication for chronic obstructive lung disease
x457581000006111	Acute interstitial emphysema
x4781471000006112	Bullous emphysema
x5516841000006119	History of chronic obstructive airway disease
x2716231000006114	Chronic obstructive lung disease
x6763251000006114	Emergency hospital admission for COPD (chronic obstructive pulmonary disease)
x7484331000006119	Optimization of medication for chronic obstructive pulmonary disease
x8235101000006114	COPD (chronic obstructive pulmonary disease) 3 monthly review
x12728161000006116	Chronic obstructive airways disease NOS
x8235131000006118	COPD (chronic obstructive pulmonary disease) 6 monthly review
x1656601000006119	COPD patient unsuitable for pulmonary rehabilitation
x909711000006111	[RFC] Chronic obstructive pulmonary disease (COPD)
x851261000006116	Chronic bronchitis, acute exac
x1780380013	Chronic obstructive pulmonary disease monitoring by nurse
x1780381012	Chronic obstructive pulmonary disease monitoring by doctor
x1488424013	Chronic obstructive pulmonary disease annual review
x457171013	Severe chronic obstructive pulmonary disease
x301450011	Chronic asthmatic bronchitis
x301451010	Chronic wheezy bronchitis
x301464011	Zonal bullous emphysema
x977901000006111	Emergency COPD admission since last appointment
x8120981000006112	COPD (Chronic obstructive pulmonary disease) patient unsuitable for pulmonary rehabilitation
x8058301000006110	COPD (chronic obstructive pulmonary disease) disturbs sleep
x9317341000006115	ACOS - asthma-chronic obstructive pulmonary disease overlap syndrome
x301448015	Mucopurulent chronic bronchitis NOS
x3011135010	Step down change in chronic obstructive pulmonary disease management plan
x424365019	Acute infective exacerbation of chronic obstructive airways disease
x2009451000006113	Chronic obstructive pulmonary disease post discharge review
x1882371000006118	Shared care chronic obstructive pulmonary disease monitoring
x1683181000000112	Chronic obstructive pulmonary disease 3 monthly review
x508562012	Chronic catarrhal bronchitis
x1488423019	Chronic obstructive pulmonary disease follow-up
x396108018	Bullous emphysema with collapse
x301453013	Acute exacerbation of chronic obstructive airways disease
x1813881000006119	On COPD (chr obstruc pulmonary disease) supportv cre pathway
x1948051000006112	Asthma-chronic obstructive pulmonary disease overlap syndrom
x301835010	[X]Other emphysema
x396110016	Other emphysema NOS
x11932331000006115	COPD GOLD group A
x8294651000006114	COPD (chronic obstructive pulmonary disease) rescue pack declined
x4510801000006114	End stage chronic obstructive pulmonary disease
x8219501000006114	Issue of COPD (chronic obstructive pulmonary disease) rescue pack
x3764031000006119	Interstitial pulmonary emphysema
x1683221000000119	Chronic obstructive pulmonary disease 6 monthly review
x2152091000000112	Has chronic obstructive pulmonary disease care plan
x2308511000000113	Shared care chronic obstructive pulmonary disease monitoring
x1823851000006119	Chronic obstructive pulmonary disease confirmed
x8287171000006115	Acute non-infective exacerbation of COPD (chronic obstructive pulmonary disease)
x301572010	Chronic emphysema due to chemical fumes
x87480013	Chronic tracheobronchitis
x1904861000006117	Chronic obstruct pulmonary disease management plan declined
x301457014	Other chronic bronchitis
x301459012	Chronic bronchitis NOS
x301836011	[X]Other specified chronic obstructive pulmonary disease
x555471000006114	Chronic obstructive airways disease
x640491000006111	Emphysema

MedCodeId	Term
x966841000006111	Chronic obstructive pulmonary disease clini management plan
x3873191000006110	Fetid chronic bronchitis
x8244871000006114	GP (general practitioner) OOH (out of hours) service notified of COPD (chronic obstructive pulmonary disease) care plan

Lewer D, *et al. BMJMED* 2022; 1:e000215. doi: 10.1136/bmjmed-2022-000215

4. Causal model for selecting confounding variables in analysis of treatment and outcomes after diagnosis of COPD

We used a causal model to select confounding variables in our analysis of treatment and outcomes after diagnosis of COPD. This model is shown in Figure 3.

Figure 3: Causal model showing how a history of illicit opioid use may affect the probability of treatment and adverse outcomes after a diagnosis of chronic obstructive pulmonary disease

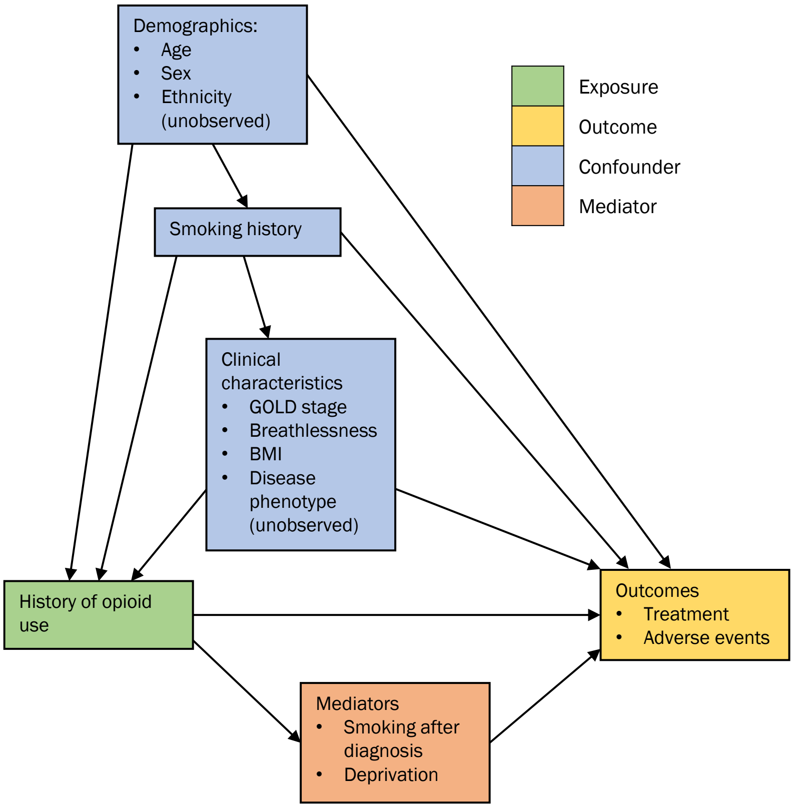

Lewer D, *et al. BMJMED* 2022; 1:e000215. doi: 10.1136/bmjmed-2022-000215

5. Characteristics of participants in the base cohort

The main article includes a table showing the characteristics of participants with a new diagnosis of COPD. Table 2 shows the characteristics of the 'base cohort'.

Table 2: Characteristics of participants in the base cohort

Variable	Level	History of using illicit opioids n (%)	Comparison group n (%)
Total		106,789 (100.0)	320,367 (100.0)
Years of follow-up (linked data)*	Median [IQR]	8.7 [4.3-13.5]	9.5 [5.0-14.4]
Years of follow-up (CPRD)*	Median [IQR]	3.2 [1.2-7.3]	5.5 [2.5-10.4]
Age at cohort entry, years	Median [IQR]	35.1 [29.0-42.3]	35.1 [29.0-42.4]
Sex	Male	73,791 (69.1)	221,373 (69.1)
	Female	32,998 (30.9)	98,994 (30.9)
Body mass index (kg/m2)	Underweight (<18.5)	5,463 (5.1)	6,827 (2.1)
	Healthy [18.5-25)	44,389 (41.6)	105,814 (33.0)
	Overweight [25-30)	20,339 (19.0)	83,682 (26.1)
	Obese [30-40)	11,127 (10.4)	48,735 (15.2)
	Severely obese (40+)	1,779 (1.7)	7,195 (2.2)
	Missing	23,692 (22.2)	68,114 (21.3)
	Median [IQR]	23.7 [21.0-27.5]	25.6 [22.7-29.3]
	Mean [sd]	24.9 [5.6]	26.6 [5.7]
Smoking at index	Never	7,261 (6.8)	146,342 (45.7)
	Ex	7,043 (6.6)	39,818 (12.4)
	Current	83,486 (78.2)	107,846 (33.7)
	Missing	8,999 (8.4)	26,361 (8.2)
Index of multiple deprivation	1 - Least deprived	7,412 (6.9)	44,051 (13.8)
	2	11,361 (10.6)	52,047 (16.2)
	3	16,339 (15.3)	57,411 (17.9)
	4	26,090 (24.4)	73,151 (22.8)
	5 - Most deprived	45,396 (42.5)	93,268 (29.1)
	Missing	191 (0.2)	439 (0.1)
Prevalent COPD		2,431 (2.3)	1,368 (0.4)
Deaths due to COPD	Number [rate per 100,000 PYs]	680 [71]	160 [6]
Incident COPD	Number [rate per 100,000 PYs]	4,016 [799]	3,051 [153]

* Follow-up for 'linked data' refers to follow-up until the final date when externally linked data (i.e. ONS mortality and Hospital Episode Statistics) is available; while follow-up for 'CPRD' refers to follow-up until the final date when primary care data (used for diagnosis of COPD) is available.
PYs = person-years

6. Definitions and exclusions for outcomes after diagnosis of COPD

Our analysis of outcomes after diagnosis of COPD (using the 'sub-cohort' 'of patients diagnosed with COPD, as described in the methods section) includes 5 treatment-related outcomes and 4 adverse outcomes. Definitions of each outcome and exclusion criteria (patients who are considered ineligible for the treatment and therefore excluded from analysis) are shown in Table 3. More detailed codelists are included in the study protocol.[1]

Table 3: Definitions and exclusion criteria for outcomes after diagnosis of COPD

Type	Outcome	Prescriptions and clinical events	Exclusion criteria (ineligible groups)
Treatment	Seasonal influenza vaccine	• Prescription of vaccine<br>• Record of vaccine given in another setting	None
	Pneumococcal vaccine	• Prescription of vaccine<br>• Record of vaccine given in another setting	Patients with a pneumococcal vaccine before diagnosis of COPD
	Pulmonary rehabilitation	• Pulmonary rehabilitation class<br>• Referral for pulmonary rehabilitation	None
	COPD-specific medication	• Inhaled corticosteroids<br>• Bronchodilators	None
	Smoking cessation support	• Referral to a specialist stop smoking team<br>• Prescription of varenicline<br>• Prescription of bupropion<br>• Prescription of nicotine replacement therapy<br>• Delivery of behavioural support	Patients who do not have records of current smoking at the time of COPD diagnosis
Adverse outcomes	Hospitalised exacerbations (based on a validated definition[6])	• Hospital admission with a primary diagnosis of COPD (ICD-10 J41-J44)<br>• Hospital admission with acute infection or acute exacerbation in any diagnosis position (J44.0 or J44.1)	None
	Unplanned hospital admissions for respiratory disease	• Hospital admission where the primary diagnosis is a respiratory disease (ICD-10 J00-J99) and the method of admission is "emergency"	None
	All-cause death	• Any death identified in linked Office for National Statistics mortality data	None
	Deaths due to respiratory disease	• Deaths identified in linked Office for National Statistics mortality data with an underlying cause of respiratory disease (ICD-10 J00-J99)	None

Lewer D, et al. BMJMED 2022; 1:e000215. doi: 10.1136/bmjmed-2022-000215

7. Sensitivity analysis of outcomes after COPD diagnosis, restricted to current smokers

We did a sensitivity analysis restricted to COPD cases with records of current smoking at diagnosis, because never-smokers may have different exposures (for example may have more genetic risk factors for COPD) and disease phenotypes, and may be more common among the comparison group. The results of this sensitivity analysis are shown in Figure 4.

Figure 4: Associations between use of illicit opioids and outcomes after COPD diagnosis, comparing the main analysis with a sensitivity analysis restricted to current smokers. Values are hazard ratios (95% CI)

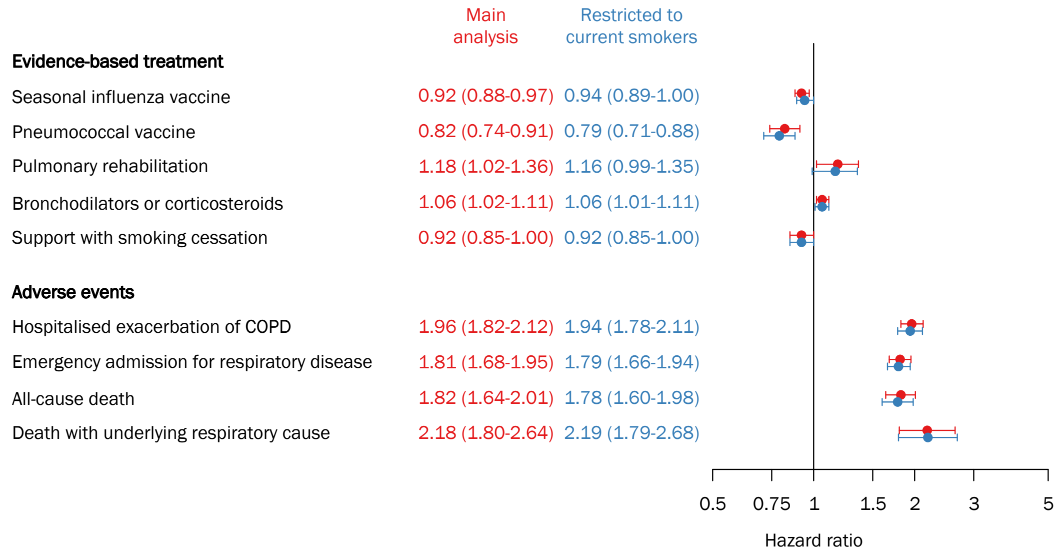

8. Detailed results of survival analysis

We used survival analysis to compare time-to-COPD-related death and time-to-COPD-diagnosis by opioid use status. The Kaplan-Meier curves are shown in Figure 5 and Figure 6, and the hazard ratios including covariates are shown in Table 4 and Table 5. We did not present the covariate coefficients in the main article because they are not the focus of this study, and the adjustment strategy may not be appropriate (ie., they may be subject to the "table 2 fallacy").

Figure 5: Kaplan-Meier estimates of the cumulative hazard of death with an underlying cause of COPD, comparing participants with and without a history of using illicit opioids (shaded area shows 95% confidence interval)

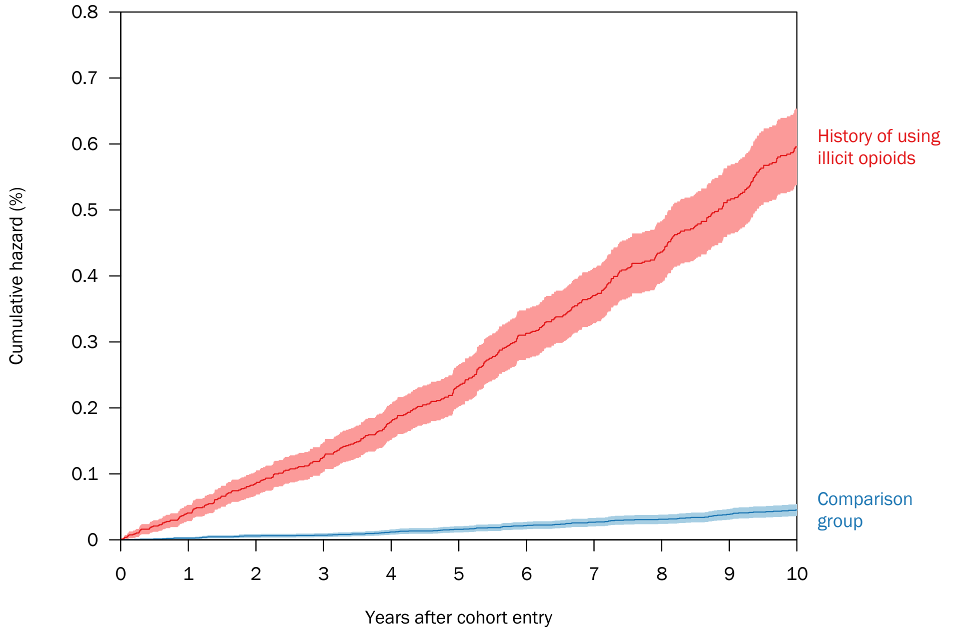

Number at risk (thousands)

Opioids	107	100	94	88	82	76	70	63	58	51	45
Comparison	320	304	290	275	258	240	223	206	188	170	152

Table 4: Hazard ratio of death with underlying cause of COPD (95% CI)

Variable	Level	Adjusted for age and sex only	Fully adjusted
History of illicit opioids		14.59 (12.28-17.33)	8.81 (7.36-10.54)
Age group	Under 25	4.26 (0.78-23.31)	4.71 (0.86-25.76)
	25-34 (ref)	1	1
	35-44	13.58 (4.98-37.03)	13.75 (5.04-37.51)
	45-54	67.67 (25.16-182.03)	74.02 (27.51-199.14)
	55-64	272.78 (101.60-732.39)	343.89 (128.03-923.68)
	65+	469.37 (173.50-1269.84)	763.62 (281.94-2068.27)
Female sex		1.03 (0.89-1.19)	1.09 (0.94-1.26)
Smoking	Never (ref)	1	1
	Ex	6.59 (4.27-10.18)	4.66 (3.01-7.22)
	Current	20.13 (13.89-29.18)	8.12 (5.55-11.90)

Figure 6: Kaplan-Meier estimates of the cumulative hazard of new COPD diagnosis, comparing participants with and without a history of using illicit opioids (shaded area shows 95% confidence interval)

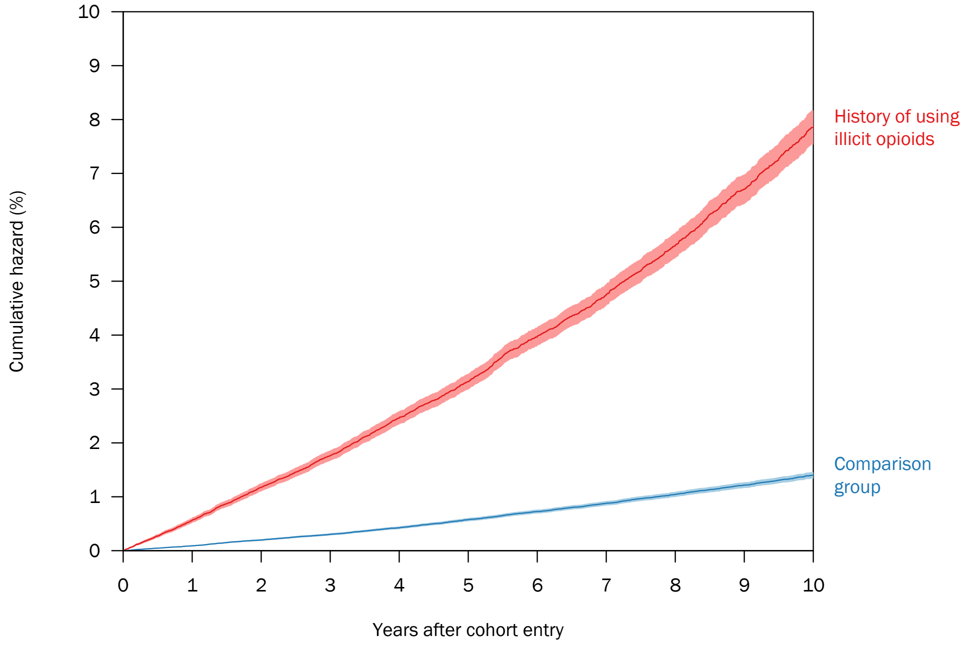

Number at risk (thousands)

	0	1	2	3	4	5	6	7	8	9	10
Opioids	102	81	66	54	45	37	31	26	22	19	16
Comparison	314	285	253	222	195	170	149	130	112	98	84

Lewer D, *et al. BMJMED* 2022; 1:e000215. doi: 10.1136/bmjmed-2022-000215

Table 5: Hazard ratio of diagnosis of COPD (95% CI)

Variable	Level	Adjusted for age and sex only	Fully adjusted
History of illicit opioids		5.89 (5.62-6.18)	3.29 (3.13-3.46)
Age group	Under 25	0.23 (0.10-0.51)	0.24 (0.11-0.54)
	25-34 (ref)	1	1
	35-44	6.10 (5.24-7.10)	6.29 (5.40-7.33)
	45-54	16.37 (14.09-19.02)	18.60 (16.01-21.61)
	55-64	29.92 (25.70-34.85)	39.16 (33.62-45.62)
	65+	30.74 (25.92-36.45)	48.19 (40.61-57.18)
Female sex		1.13 (1.07-1.18)	1.27 (1.21-1.34)
Smoking	Never (ref)	1	1
	Ex	4.02 (3.56-4.55)	3.52 (3.11-3.98)
	Current	13.97 (12.68-15.39)	9.00 (8.14-9.94)

Lewer D, *et al. BMJMED* 2022; 1:e000215. doi: 10.1136/bmjmed-2022-000215

9. References for supplementary information

1 Lewer D, Denaxas S, Gonzalez-Izquierdo A, *et al.* Incidence of COPD and quality of subsequent treatment among people with a history of using illicit opioids: a cohort study in England (PROTOCOL). 2021.https://doi.org/10.5522/04/14207687.v1

2 Pedersen A, Mikkelsen E, Cronin-Fenton D, *et al.* Missing data and multiple imputation in clinical epidemiological research. *Clin Epidemiol* 2017;**Volume 9**:157–66. doi:10.2147/CLEP.S129785

3 Greenland S, Finkle WD. A Critical Look at Methods for Handling Missing Covariates in Epidemiologic Regression Analyses. *Am J Epidemiol* 1995;**142**:1255–64. doi:10.1093/oxfordjournals.aje.a117592

4 Ohneberg K, Beyersmann J, Schumacher M. Exposure density sampling: Dynamic matching with respect to a time-dependent exposure. *Stat Med* 2019;**38**:4390–403. doi:10.1002/sim.8305

5 Quint JK, Mullerova H, DiSantostefano RL, *et al.* Validation of chronic obstructive pulmonary disease recording in the Clinical Practice Research Datalink (CPRD-GOLD). *BMJ Open* 2014;**4**:e005540–e005540. doi:10.1136/bmjopen-2014-005540

6 Rothnie KJ, Müllerová H, Thomas SL, *et al.* Recording of hospitalizations for acute exacerbations of COPD in UK electronic health care records. *Clin Epidemiol* 2016;**Volume 8**:771–82. doi:10.2147/CLEP.S117867
